# Evaluation of Tissue Metabolites with High Resolution Magic Angle Spinning MR Spectroscopy Human Prostate Samples After Three-Year Storage at −80 °C

**Published:** 2007-04-18

**Authors:** Kate W. Jordan, Wenlei He, Elkan F. Halpern, Chin-Lee Wu, Leo L. Cheng

**Affiliations:** 1 Departments of Pathology and; 2 Radiology Massachusetts General Hospital, Harvard Medical School Boston, Massachusetts

**Keywords:** High resolution magic angle spinning, magnetic resonance spectroscopy, human prostate, metabolites, tissue frozen storage

## Abstract

Accurate interpretation and correlation of tissue spectroscopy with pathological conditions requires disease-specific tissue metabolite databases; however, specimens for research are often kept in frozen storage for various lengths of time. Whether such frozen storage results in alterations to the measured metabolites is a critical but largely unknown issue. In this study, human prostate tissues from specimens that had been stored at −80 °C for 32 months were analyzed with high resolution magic angle spinning (HRMAS) magnetic resonance (MR) spectroscopy, and compared with the initial measurements of the adjacent specimens from the same cases when snap frozen in the operation room and kept frozen for less than 24 hours. Results of the current study indicate that that the storage-induced metabolite alterations are below the limits that tissue MR spectroscopy can discriminate. Furthermore, quantitative pathology evaluations suggest the observed alterations in metabolite profiles measured from the adjacent specimens of the same prostates may be accounted for by tissue pathological heterogeneities and are not a result of storage conditions. Hence, these results indicate that long-term frozen storage of prostate specimens can be quantitatively analyzed by HRMAS MR spectroscopy without concerns regarding significant metabolic degradation or alteration.

## Introduction

Since the introduction of the high resolution magic angle spinning (HRMAS) methodology to investigate biological tissue samples (Cheng et al. 1996), a concern frequently posed has been the possible effects of sample storage duration on the measured metabolite concentrations. This concern is scientifically logical and clinically relevant because of the increasing tests and evaluations of the HRMAS spectroscopy methodology for its biomedical utilities and the observed correlations between the measured tissue metabolite changes and their underlying pathological alterations.

A large body of research has suggested the potential utility of using tissue metabolic profiles thus defined to indicate and predict the existence of certain pathological conditions (Swanson et al. 2003; Sitter et al. 2004; Tugnoli et al. 2004; Cheng et al. 2005; Duarte et al. 2005; Keshari et al. 2005; Tugnoli et al. 2005; Sitter et al. 2006). Therefore, the promising and practical utilities of this methodology in disease diagnosis, patient prognostication, and therapy monitoring have been progressively recognized.

We realize that as interest in disease-related HRMAS MR spectroscopy grows the HRMAS methodology is often being tested at a research stage. As such, more often then not, human specimens of interest from surgeries and/or biopsies, or even from autopsies, are used. However, most of these specimen cannot be analyzed immediately without frozen storage durations due to various technical, administrative, or logistical reasons. Very often research projects, particularly retrospective studies, also use frozen human samples, such as those collected by various tumor banks, stored in either liquid nitrogen (−196 °C) or −80 °C freezers for months or even years. At present there are no data to either support or contradict the rationale for using these samples, as the existence or non-existence of frozen storage induced metabolic alternations is largely unknown.

Here, we report a study that we designed to measure prostate tissue metabolite profile for samples kept at −80 °C during a storage period of three years. In this study, we used a set of well-characterized human prostate tissue specimens from prostatectomies of cancer patients. In 2002, in order to evaluate tissue freeze-thawing processes via the measured metabolite concentrations, we collected and analyzed 12 fresh human prostate specimens from five patients and reported those results in an article in 2003 (Wu et al. 2003). After that study, the extra specimens were in frozen storage at −80 °C from 2002 to 2005.

## Experimental

### Tissue protocol

MR spectroscopic analyses of surgical specimens from human prostates were approved by the Institutional Review Board (IRB) at Massachusetts General Hospital. Twelve human prostate specimens were collected in 2002 in the operating room from different prostatic zones (central, transitional and peripheral) of five prostatectomy cases. Among these 12 specimens, 11 specimens left remaining tissue samples after the 2002 study (Wu et al. 2003). These samples have been stored at −80 °C from July 2002 to March 2005. For the current study, a total of 15 samples cut from these 11 specimens were analyzed with spectroscopy (duplicates were measures from four samples with extra material).

### HRMAS proton NMR

The spectroscopic experimental protocol is exactly the same as used in the 2002 study. Briefly, MR experiments were carried out on a Bruker (Billerica, MA) AVANCE spectrometer operating at 600 MHz (14.1T). A 4 mm zirconia rotor was used with Kel-F plastic inserts which created a spherical sample space of ~10 μl located at the center of the detection coil. A small (~0.1 mg) silicone rubber sample was permanently fixed inside one of the Kel-F spacers, positioned within the detection coil but not in contact with the sample, which functioned as an external standard for both frequency reference (0.06 ppm from TMS) and concentration quantification. Approximately 1.0 μl of D_2_O was added into the rotor with the tissue sample for ^2^H field locking. All spectroscopy measurements were carried out at 3 °C for better tissue preservation. The rotor-spinning rate was regulated by a MAS controller (Bruker), and verified by the measurement of inter-SSB distances from spectra, with an accuracy of ±1.0 Hz. A repetition time of five seconds and 32 transients were used to acquire each spectrum.

Spectra were collected with spinning rates of 600 and 700 Hz, with or without a rotor synchronized DANTE sequence (1000 DANTE pulses of 1.5 μs, 8.4° flip angle) (Taylor et al. 2003). A rotor-synchronized CPMG filter (10 ms) was included in the pulse sequence after the execution of the DANTE frequency-selective pulses to reduce broad resonances associated with probe background, rotor, and/or macromolecules. Spectra measured at 600 Hz spinning without DANTE were used to quantify the total metabolite signal intensity including tissue water, its sidebands, and all the metabolites.

Spectroscopic data were processed with Nuts software (Acorn NMR Inc. Livermore, CA) according to the following procedures. All free induction decays were subjected to 1Hz apodization before Fourier transformation, baseline correction, and phase adjustments of both zero and first order. Resonance intensities reported here represent integrals of curve-fittings with Lorentzian-Gaussian line-shapes. All spectra were processed manually and objectively, as in the 2002 study, without knowledge of tissue pathological information. As previously reported, resonance intensities, depending on the particular spectral regions, were analyzed from one of the two spectra where there was no effect of water spinning sidebands (SSB) and DANTE suppression (Taylor et al. 2003). The absolute concentration for a metabolite was estimated according to the metabolite intensity measured in DANTE spectra, the total MR spectral signal intensity from the single pulse measurement, and the intensities of the rubber standard measured under both conditions, according to the formula below:

$$\begin{array}{l} {}[M]={\left[(I_{M}/n)/I_{STD}\right]}_{\text{w}/{\text{oH}}_{2}O}{\left[I_{STD}/I_{H_{2}O/2}\right]}_{H_{2}O}\\ {}*55.56\times 10^{3}(\mu \text{mol}/\text{g}) \end{array}$$

where **I***_M_* represents measured intensities for metabolites, **I***_STD_* is the measured intensity of the external rubber reference, *n* is the number of protons giving rise to the resonance, ***I***_H_2_O/2_ is the intensity of water, and 55.56 × 10^3^ (μmol/g) is the concentration of water.

### Histopathology

After spectroscopy analyses all 15 samples were fixed in formalin for histopathology evaluations. Fixed tissue samples were embedded in paraffin, cut into 5 μm sections, and stained with Hematoxylin and Eosin. Sets of serial-sections cut 100 μm apart were obtained from each sample. Volume percentages of histological features were quantified from these histopathological images (Burns et al. 2004).

## Results and Discussions

**Figure 1:**
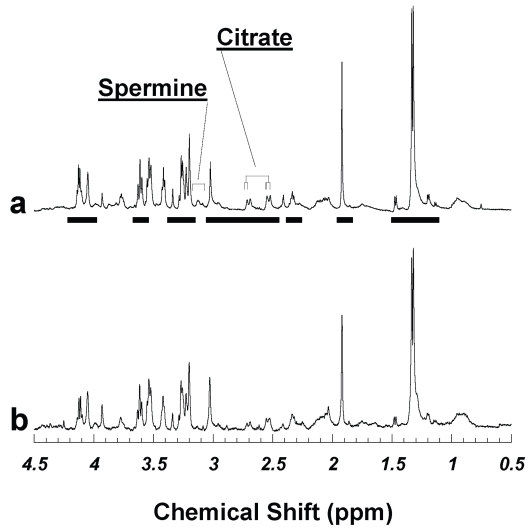
Visually undifferentiated human prostate tissue HRMAS proton spectra from two cuts of the same surgical specimen of a cancerous prostate measured (a) in 2005 after being stored at −80 °C for 32 months, and (b) in 2002 when the sample was thawed after being frozen overnight. Quantitative pathology detected no histopathologically identifiable cancerous glands in either sample; other than stromal cells, the majority of prostate pathology in both samples was histopathologically benign epithelia, which comprised 46.1 and 33.8%, for (a) and (b), respectively. Figure 1 (b) was adopted from Figure 1 (b) of Ref. (Wu et al. 2003). Metabolite intensities analyzed in the current study are labeled with horizontal bars under spectrum 1a.

Figure 1 compares two proton HRMAS spectra acquired from the adjacent specimens of the same prostate after tissue samples were frozen at −80 °C either for less than 24 hours in July 2002 (Fig. 1b, same as presented in Figure 1b in the previous report (Wu et al. 2003)), and for more than 32 months until March 2005 (Fig. 1a). The tissue metabolite profiles in these spectra showed little difference, suggesting that discriminating the possible metabolite degradations over a tissue −80 °C storage period up to 32 months may be below the capability of current analyses. However, identical prostate metabolite profiles obtained 32 months apart from adjacent samples of the same prostate were not observed with every pair tested.

To investigate the discrepancy, as the HRMAS methodology allows post - acquisition histopathological evaluations, we performed quantitative pathology evaluations on the measured samples. Histopathological examinations of serial sections produced from these tissue samples after their spectroscopy analyses indicated that among all the tested samples there were no cancerous glands present. This was not surprising given the heterogeneous nature of the prostate and the fact that only 10% of research samples were found to have histologically positive cancer glands (Cheng et al. 2005). Hence, the major quantifiable histopathological differences among the current research samples were the variations in the volume percentage ratios between histopathologically benign prostate epithelial glands and stromal cells (Table 1). Quantitative histopathology results showed that the amounts of benign epithelia were (a) 46.09 and (b) 33.75% (vol) in Figure 1 for the two samples measured for in 2005 and 2002, and the relative percentage difference was about 15% (= (46.09 − 33.75)/(46.09 + 33.75)), as shown in Table 1. This table reflected heterogeneity in pathological compositions between adjacent tissues of the same prostate for the 15 pairs of samples reported. In this table we summarize all 15 tested samples and their counter measurements evaluated in 2002, together with the calculated pathological absolute differences represented by benign epithelial percentages (Diff. Epith. %), and the relative differences in terms of the absolute differences normalized by the sum of the values of the both years.

Based on observations obtained with sample pairs represented by Figure 1, we hypothesize that spectroscopic differences observed between samples measured in 2002 and 2005 were predominately caused by variations in pathological composition. We tested this hypothesis in Table 2, which compares the evaluated paired t-tests results of the 13 most intense resonance peaks measured from the HRMAS spectra for all 15 sample pairs with seven pairs with relative percentage differences of histological features less than 20%. If a statistically significant difference for a particular metabolite existed between the two tested groups, i.e. groups measured in 2002 *vs*. those in 2005, a paired t-test can be used to detect this existence. However, if the resulting p-value is greater than 0.05 (after a Bonferroni correction for multiple comparisons), it would indicate that either there is no statistically significant difference between the two groups of interest, or the test does not have enough statistical power to reveal the difference. In Table 2, some metabolites, such as creatine (Cr, 3.03 ppm) and citrate (Cit, 2.70 – 2.73 ppm), displayed seemingly significant differences (even after Bonferroni corrections) between the two groups when considering all 15 sample pairs. These significances do not persist when more restrictive controls on the allowable variations in pathological compositions are applied by including only sample pairs of the relative pathological differences <20%.

**Figure 2:**
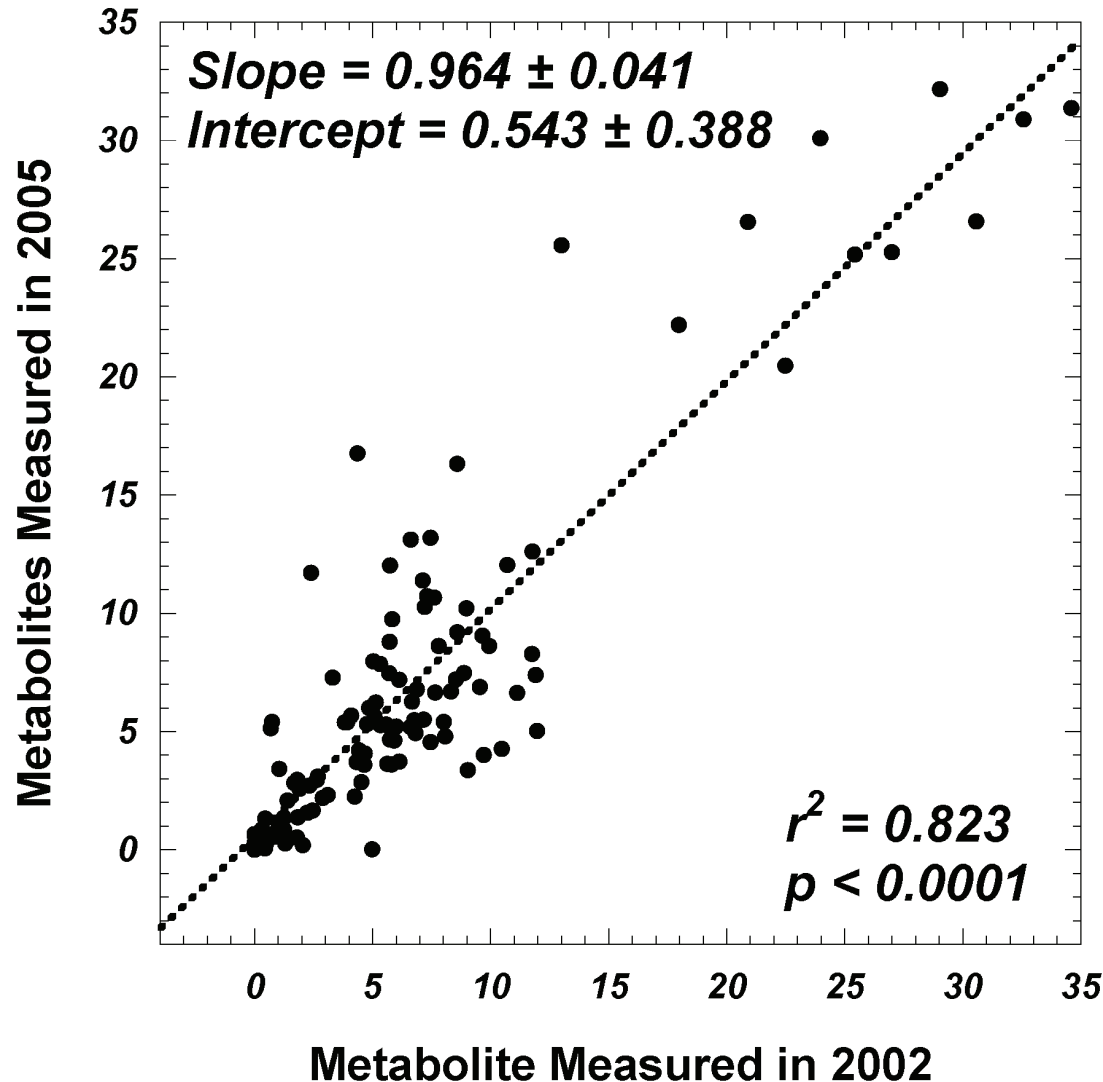
A linear correlation between metabolite intensities (normalized by total spectral intensity excluding tissue water signals) measured in 2002 with those measured in 2005 for seven pairs of tissue samples; within each pair the changes in volume percentages of histological features are <20%.

Considering the subgroup of seven tissue sample pairs with less than 20% relative pathological variations within each pair, Figure 2 plots the relationship of relative metabolite intensities (resonance peaks normalized by the total spectral intensity excluding tissue water signals) measured from 2002 spectra against those obtained in 2005. The statistically significant linear relationship between the two data sets, with slope equal to unity and intercept close to 0, indicates the absence of tissue metabolite changes after frozen storage of 32 months.

**Figure 3:**
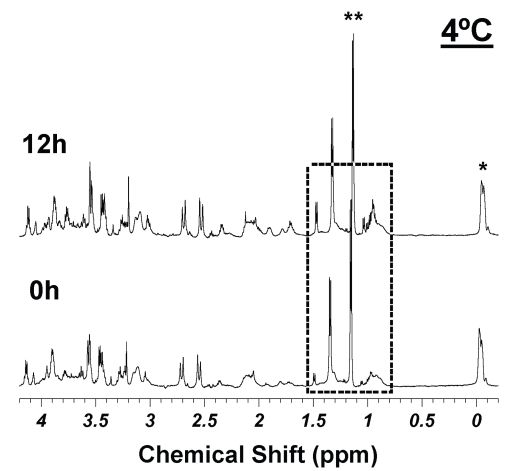
Intact prostate tissue HRMAS spectra measured at 4°C at 0 and 12 hours. *denotes an external rubber standard; **indicates alcohol contaminations. Comparison between these two spectra reveals metabolite products due to tissue degradations. Readers are instructed to pay special attention to the boxed-in spectral regions, where the increased intensities of free amino acids such as alanine (1.48 ppm), valine (0.96 ppm) etc. are clearly visible in the 12 h spectrum.

Following the analytic procedures used for the 2002 report (Wu et al. 2003), in Table 3, we further examined concentrations of 21 prostate metabolites summarized in the 2002 report for seven sample pairs of relative differences of pathological volume percentages less than 20% within each pair (in Table 1). Metabolite concentrations obtained in 2002 are compared with those measured in 2005, together with their respective p values of paired t-tests included. Also included in this table are the power calculations to determine the levels of Type II errors, if any, for each measured metabolite. Specifically, based on the standard deviations measured with the seven-pairs of samples, using two-sided evaluation of a 5% significant level, and at an 80% power level, the minimal detectable differences in metabolite concentrations for each metabolites are presented in the table as the percentage of the 2002 values. For instance, the detectable difference 38.9% in the first row of the table for lactate indicates that based on the current study we can conclude there is no lactate change measured in 2005 that is greater than 38.9% of its value in 2002, i.e. either less than 7.86 mM or greater than 17.88 mM. Similarly, for phosphocholine (Pch, 3.22 ppm), we can conclude that there are no changes that are greater than 95.1% of its 2002 value; and for choline (3.20 ppm), no changes detected that are larger than 67.1%. Of a particular interest, we can confidently conclude that with alanine our data indicate that no changes measured in 2005 are greater that 75.7% of the 2002 values. This is very important evidence supporting our hypothesis that no MR spectroscopy visible tissue degradation occurs during long term storage; as with our tissue MR spectroscopy experience, alanine and other free amino acids are the first metabolites that present due to the break down of proteins, as shown in Figure 3. Comparing spectra in Figure 3, particularly in the boxed-in region, with the corresponding regions in spectra in Figure 1, it is not difficult to recognize the metabolite changes resulting from tissue degradations at 4 °C after 12 hours under the 3.6 kHz HRMAS experimental condition.

There are a number of related issues that are worthy of discussion. First, examination of Table 1 reveals that although our data analysis made the best effort to reduce the influence of pathological heterogeneity by grouping the seven sample pairs having relative differences <20%, this difference still varied from 2.16% to 15.46%. More importantly, the absolute epithelial volume percentage in the analyzed samples varied from 8.49% to 46.09%. This difference undoubtedly contributed to the increase in the calculated standard deviations, which in turn affect the estimations of the minimal detectable limits. However, these confounding factors of bio-diversity and tissue heterogeneity are intrinsically associated with clinical studies and cannot be extricated, although special considerations may be applied to reduce the effects. Secondly, instead of investing in rigorous efforts to reduce the confounding factors in order to measure the true values of storage related tissue metabolite differences, if in existence, one may wish to consider there are acceptable levels of uncertainty, as long as they does not interfere with the clinical significance of the resulted metabolite profiles (Cheng et al. 2005).

Combining data in Table 3, which sets the boundary for the possible existence of type II errors associated with each analyzed metabolite, with Figure 2 that indicates the overall preservation of tissue metabolites during storage, we can conclude that there seem to be no HRMAS-quantifiable statistically significant prostate metabolite differences that can be contributed to sample storage at −80 °C for 32 months. We have also noted that some standard deviations in Table 3 are greater than the mean; this is due to the small sample number (n = 7) and the skewed concentration distributions that deviated away from a normal bell curve distribution.

However, we wish to emphasize that the above conclusion regarding a lack of significant measurable metabolite changes over long term frozen storage can only be utilized within the current experimental conditions. For instance, since the tested prostate specimens contained no histologically visible cancer glands, theoretically, we cannot simply extend the experimental observations directly to cancer cells. However, based on observations here reported, we may suggest that quantification of prostate pathologies may be more critical for the correct interpretation of tissue spectroscopy results than any possible storage effects. Furthermore, although the concept that storage of tissue samples at −80 °C halts the processes of metabolite pathways may be applicable to other types of tissues, the universal applicability is not self-evident and cannot be directly extended from the current results measured on prostate tissues. Separate studies on the types of tissues of interest are necessary to verify the concept.

## Conclusion

Analyzing and comparing human prostate tissue spectra from specimens that have been stored at −80 °C for 32 months after their initial spectroscopy measurements when snap frozen for less than 24 hours has led us to conclude that the possible frozen storage induced metabolite alterations are as minimal as tissue MR spectroscopy can distinguish. Such alterations, even if in existence, are much less critical to the interpretation of tissue HRMAS spectroscopy for pathological purposes than the influence of innate pathological heterogeneities.

## Figures and Tables

**Table 1:** Quantitative pathology results of prostate sample pairs measured in 2002 and 2005. No histopahtologically identifiable cancer glands were detected in these samples, and the major pathological components were histologically benign epithelia and stroma. The quantitative results are presented as the percentage of benign epithelia as Epith. 2002 (% Vol) and Epith. 2005 (%Vol), respectively. Diff. Epith. %: the absolute values of the epithelial difference between each sample pair. The relative difference is presented as the ratio of the absolute difference over the sum of epithelial percentages for each pair; (a) two values indicate specimens were analyzed twice in 2005; (b) Bold identifies samples with values <20% of relative epithelial differences.

Specimen No.	Epith. 2002 (% Vol)	Epith. 2005 (% Vol)	Diff. Epith. %	|Epith. 2002 − Epith. 2005| (Epith. 2002 + Epith. 2005)%
1	4.01	46.98, 11.69^a^	42.97, 7.68	84.27, 48.92
2	3.49	32.60, 23.28	29.11, 19.79	80.66, 73.93
3	3.84	14.88	11.04	58.97
4	26.64	24.61	2.03	3.96^b^
5	38.31	40.00	1.69	2.16
6	0.00	16.11, 4.74	16.11, 4.74	100.00, 100.00
7	27.76	33.12	5.36	8.80
8	23.67	18.89, 18.30	4.78, 5.37	11.23, 12.79
9	8.69	8.49	0.20	1.164
10	33.75	46.09	12.34	15.46
11	10.00	41.19	31.19	60.93

**Table 2:** The p-values of paired t-tests for the 13 most intense resonance peaks measured from the HRMAS spectra. Based on the principle of Bonferroni correction to account for the possible existence of type I error, a p-value of <0.0038 represents statistical significance.

	AllSamples (n = 15)	|Epith. 2002 − Epith. 2005| (Epith. 2002 + Epith. 2005) <20% (n = 7)
Lac(4.10–4.14)	0.0092	0.1661
MI(4.05)	0.5567	0.3429
3.29	0.0234	0.0690
3.27	0.8176	0.2990
3.25–3.26	0.1066	0.6920
Pch(3.22)	0.2646	0.8499
Chol(3.20)	0.0560	0.2975
Spm(3.05–3.14)	0.2023	0.1431
Cr(3.03)	0.0027	0.0120
Cit(2.70–2.73)	0.0042	0.0734
Acet(1.92)	0.0724	0.2857
Ala(1.47–1.49)	0.6829	0.5520
Lac(1.32–1.34)	0.3878	0.8709
Mean	0.2451	0.3580
Standard Dev	0.2807	0.2936

**Table 3:** The concentrations of 21 prostate metabolites for seven sample pairs with relative differences of epithelial volume percentages less than 20% within each pair. Metabolite concentrations (means and standard deviations) obtained in 2002 are compared with those measured in 2005 together with their respective p-values (without Bonferroni corrections) of paired t-tests. Also included in this table are the power calculations to determine the levels of Type II errors for each measured metabolite. These calculations are based on the standard deviations measured with the seven-pairs of samples, using two-sided evaluation of a 5% significant level, and at an 80% power level to determine the minimal detectable differences in metabolite concentrations for each metabolites and presented in the table as the percentage of the 2002 values. These 21 metabolites included all the reported metabolites in Ref. (Wu et al. 2003).

	2005		2002		Paired t-Test	Detectable Diff (%)
	Mean	SD	Mean	SD		
Lac(4.10–4.14)^a^	14.29	3.98	12.87	6.68	0.38	38.9
MI(4.05)	10.30	2.77	12.57	5.39	0.31	54.4
3.60–3.63^b^	16.98	6.78	20.17	13.57	0.56	78.8
3.34	5.18	2.08	5.49	1.95	0.82	61.9
3.29	1.11	0.63	2.53	2.13	0.13	108
3.27	8.79	2.38	11.17	5.60	0.30	62.8
3.25–3.26	12.22	3.32	13.95	5.85	0.26	33.8
Pch(3.22)	1.05	0.28	1.44	1.05	0.38	95.1
Chol(3.20)	1.53	0.22	1.70	0.91	0.64	67.1
Spm(3.05–3.14)	1.75	2.69	2.21	1.72	0.52	103
Cr(3.03)	2.66	0.92	5.19	3.69	0.08	76.3
Cit(2.70–2.73)	4.87	1.86	3.44	1.66	0.19	94.4
2.31–2.37	5.60	2.13	10.17	3.32	0.06	48.1
2.01–2.14	29.67	14.92	42.51	6.99	0.13	47.3
Acet(1.92)	1.36	3.02	1.19	2.54	0.44	58.8
1.68–1.78	10.89	7.11	10.98	7.34	0.98	124
Ala(1.47–1.49)	1.00	0.42	1.52	1.13	0.18	75.7
Lac(1.32–1.34)	17.45	4.18	20.53	9.80	0.37	51.6
1.19–1.20	2.70	2.30	2.13	2.67	0.35	91.1
1.04–1.05	0.51	0.32	1.59	2.21	0.25	183
Lipid(0.90)^c^	22.87	5.11	14.65	6.55	0.03	84.6

## Abbreviations

HRMAS: high resolution magic angle spinning
MR: magnetic resonance
CPMG: Carr-Purcell-Meiboom-Gill
